# Childhood obesity and allergic rhinitis: A meta-analysis

**DOI:** 10.17305/bb.2025.12982

**Published:** 2025-09-24

**Authors:** Xinxin Xing, Sihao Zhu, Guang Zhou, Yubo Ma, Hai Wang

**Affiliations:** 1 Department of Pediatrics II, The First Affiliated Hospital of Heilongjiang University of Chinese Medicine, Harbin, China

**Keywords:** Allergic rhinitis, obesity, children, risk factor, meta-analysis

## Abstract

Allergic rhinitis (AR) is a prevalent chronic condition in childhood, and its increasing incidence has prompted research into potential associations with modifiable factors such as obesity. This meta-analysis aimed to assess the multivariate-adjusted relationship between childhood obesity and AR. A systematic search was conducted across PubMed, Embase, and Web of Science for observational studies that reported on the association between obesity and AR in children. Only studies that included multivariate adjustments for at least age and sex were considered. Random-effects models were employed to pool odds ratios (ORs) with 95% confidence intervals (CIs), accounting for heterogeneity. Fifteen cross-sectional studies comprising 23 datasets involving a total of 569,856 children were included in the analysis. The overall results indicated that obesity was not significantly associated with AR (adjusted OR: 1.04, 95% CI: 1.00–1.09; *P* ═ 0.08; *I*^2^ ═ 24%). However, subgroup analyses revealed a significant association in Western countries (OR: 1.12, 95% CI: 1.00–1.24; *P* ═ 0.04; *I*^2^ ═ 0%), while no significant association was found in Asian countries (OR: 1.04, 95% CI: 0.97–1.12; *P* ═ 0.27; *I*^2^ ═ 52%). Notable associations were identified in studies utilizing national or international BMI cutoffs (OR: 1.06, 95% CI: 1.01–1.10; *P* ═ 0.02) and those with physician-diagnosed AR (OR: 1.07, 95% CI: 1.02–1.13; *P* ═ 0.006), but not in studies employing the 95th percentile BMI definition or ISAAC-based AR diagnosis. No significant differences were observed based on age or sex. Meta-regression analysis indicated that age, sex, and study quality score did not significantly influence the results (*P* all > 0.05). Egger’s test revealed no evidence of publication bias (*P* ═ 0.43). In conclusion, while no significant overall association between childhood obesity and AR was found, subgroup analyses suggest potential links within specific populations and under particular methodological definitions. These findings should be interpreted with caution, and further longitudinal studies are necessary to determine whether preventive strategies aimed at reducing childhood obesity may also impact allergic outcomes.

## Introduction

Allergic rhinitis (AR) is a common chronic inflammatory disorder of the upper airway in children, characterized by symptoms such as sneezing, nasal congestion, rhinorrhea, and nasal itching, often accompanied by ocular manifestations [1, 2]. Globally, AR affects up to 40% of children, with its prevalence increasing notably in urbanized and industrialized areas [3, 4]. Although AR is not life-threatening, it significantly impairs children’s quality of life, leading to sleep disturbances, decreased cognitive performance, increased school absenteeism, and behavioral issues [5, 6]. Furthermore, AR frequently coexists with other atopic conditions such as asthma and eczema, thereby exacerbating its clinical effects [7]. While genetic predisposition is a critical factor, environmental influences, including air pollution, secondhand smoke, and socioeconomic status, also contribute to the development of AR [8, 9]. Identifying modifiable risk factors for AR is essential for early prevention and for mitigating long-term health implications in children.

Obesity, defined as a body mass index (BMI) at or above the 95th percentile for age and sex, has emerged as a significant concern in the pediatric population, affecting over 340 million children and adolescents globally [10, 11]. Childhood obesity is linked to adverse physical and psychosocial outcomes and often persists into adulthood, heightening the risk of chronic diseases such as type 2 diabetes and cardiovascular disorders [12, 13]. Recent studies have suggested a potential link between obesity and allergic diseases. Adipose tissue functions as an endocrine organ, releasing pro-inflammatory cytokines and adipokines that may promote systemic inflammation, disrupt T-helper cell balance, and compromise the integrity of the epithelial barrier in the airways, thus increasing susceptibility to allergic reactions [14, 15]. Despite this biological plausibility, epidemiological evidence regarding the relationship between obesity and AR in children has been inconsistent. Some studies indicate a positive association [16, 17], while others find no significant link [18–30], likely due to variations in population characteristics, definitions of obesity, or methods for diagnosing AR. Notably, childhood obesity can be defined using age- and sex-specific BMI thresholds above the 95th percentile or fixed international BMI cut-offs [11], which may encompass different populations and account for variability in findings across studies. To address these uncertainties, we conducted a systematic review and meta-analysis to evaluate the association between obesity and AR in children and to examine potential effect modifiers through predefined subgroup analyses.

## Materials and methods

This study adhered to the PRISMA 2020 guidelines [31, 32] and the Cochrane Handbook [33] for systematic reviews and meta-analyses, encompassing study design, data collection, statistical methods, and result interpretation. Additionally, the protocol was registered with PROSPERO under ID CRD420251108821. The completed PRISMA 2020 checklist is available in Supplemental file 1.

### Database search

To identify studies relevant to this meta-analysis, we conducted a comprehensive search of the PubMed, Embase, and Web of Science databases, employing a wide range of search terms. This included the following combined terms: (1) “obesity” OR “obese” OR “overweight” OR “body mass index” OR “BMI”; (2) “allergic rhinitis” OR “atopic rhinitis” OR “allergic rhinitides” OR “atopic rhinitides”; and (3) “children” OR “pediatric” OR “paediatric” OR “adolescents.” The search was restricted to studies involving human subjects and included only full-length articles published in English in peer-reviewed journals. Grey literature was excluded due to its lack of peer review, which may compromise the reliability of findings. Additionally, we manually reviewed the references of related original and review articles to identify other relevant studies. The search encompassed all records from the inception of the databases up to May 26, 2025. The detailed search strategy for each database is outlined in Supplemental file 2.

### Study eligible criteria

We utilized the PECO framework to establish the inclusion criteria:

P (patients): Children and adolescents under the age of 18 years. Eligible participants included individuals from the general pediatric population or specific subgroups (e.g., school-aged children and adolescents) who have been assessed for both body weight status (obesity) and AR.

E (exposure): The exposure under review in this meta-analysis was childhood obesity, defined using standardized criteria such as a BMI at or above the 95th percentile for age and sex, or equivalent fixed cut-off values based on national or international references. Only studies that distinctly categorized and reported on obesity were included.

C (comparison): The comparator group in the meta-analysis consisted of children with normal weight, defined according to BMI criteria as falling below the 85th percentile for age and sex or within the normal BMI range based on national or international growth references.

O (outcome): The primary outcome of interest was the presence of AR in children, determined by physician diagnosis, validated questionnaires (e.g., the International Study of Asthma and Allergies in Childhood [ISAAC] criteria), or self-reported diagnoses corroborated by symptoms. Only studies reporting multivariate-adjusted associations between obesity and AR in children were included, accounting for at least age and sex.

S (study design): We included observational studies, such as cohort studies, case-control studies, and cross-sectional studies.

We excluded reviews, editorials, other meta-analyses, studies involving adult populations, studies focusing solely on overweight or combining overweight with obesity, studies not reporting AR as an outcome, and studies providing only univariate data. In cases of overlapping populations, we included the study with the largest sample size in the meta-analysis.

### Study quality evaluation

Two authors independently conducted the literature search, study selection, quality assessment, and data extraction. Disagreements were resolved through discussion with the corresponding author. The quality of the studies was assessed using a modified version of the Newcastle–Ottawa Scale (NOS) adapted for cross-sectional studies [34], as applied in previous meta-analyses [35, 36]. The assessment rubric included selection (4 items), comparability (2 items), and outcome (3 items), with a maximum score of 9 points. Detailed scoring criteria are provided in Supplemental file 3. Studies scoring ≥7 were deemed high quality.

### Data collection

The data collected for analysis included study details (author, year, study country, and design), participant characteristics (source of the children, age range, mean age, and proportion of boys), the definition and number of children with obesity, methods for diagnosing AR, the number of children with AR, and covariates adjusted in the regression models.

### Statistical analysis

We utilized odds ratios (ORs) and 95% confidence intervals (CIs) to evaluate the association between obesity and AR in children, comparing those with obesity to their normal-weight counterparts. ORs and standard errors were either directly extracted or calculated from 95% CIs or *P* values, then log-transformed to stabilize variance and normalize the data [33]. In instances where multiple ORs were reported from different models, we selected the model with the most comprehensive adjustments.

Heterogeneity was assessed using the Cochrane *Q* test and *I*^2^ statistic [37], where a *P* value of < 0.10 indicated significant heterogeneity. *I*^2^ values were interpreted as follows: <25% indicated low heterogeneity, 25%–75% indicated moderate heterogeneity, and >75% indicated high heterogeneity. A random-effects model was employed to pool the data, addressing the heterogeneity across studies [33]. The primary analyses utilized the DerSimonian–Laird (DL) method, while sensitivity analyses were performed using the restricted maximum likelihood (REML) estimator [33].

**Figure 1.**
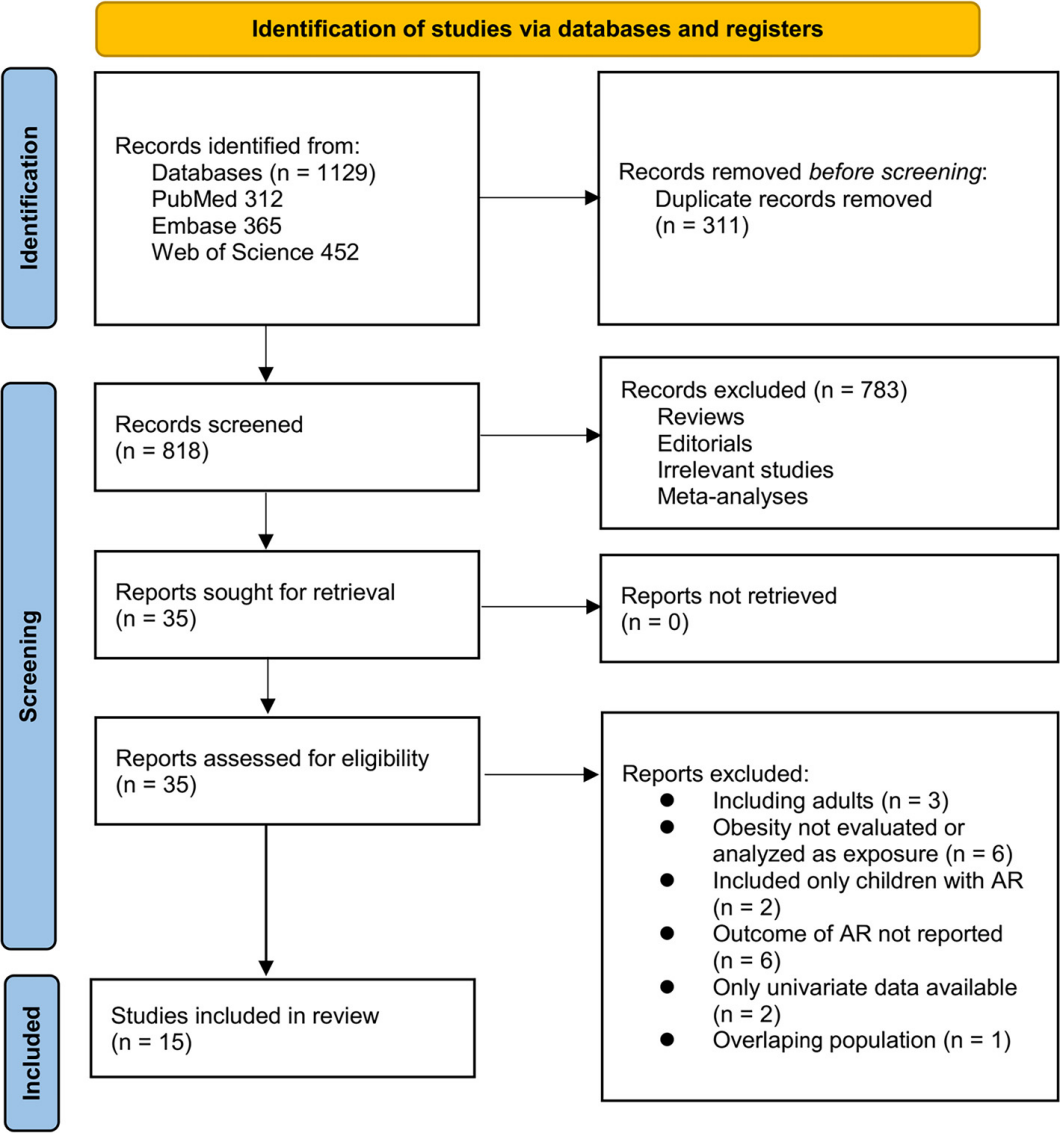
Flowchart of database search and study inclusion.

Additionally, we conducted a sensitivity analysis by combining sex-stratified datasets into a single effect size per study to mitigate potential within-study clustering. A further sensitivity analysis was executed by excluding one dataset at a time to assess the robustness of our findings [33]. Predefined subgroup analyses were performed based on the countries of the studies (Asian vs Western), average age of the children, sex distribution, definitions of obesity (national/international BMI cutoffs vs BMI ≥ the 95th percentile for age and sex), methods for diagnosing AR (ISAAC criteria or physician diagnosis), and NOS scores of the included studies. Medians of continuous variables were used to evenly divide the subgroups.

Subsequently, a univariate meta-regression analysis was conducted to determine if mean age, proportion of males, or NOS scores influenced the association between obesity and AR [37]. Publication bias was assessed using funnel plots, with visual inspection for asymmetry, alongside Egger’s test [38]. The certainty of the evidence was evaluated through the Grading of Recommendations Assessment, Development, and Evaluation (GRADE) approach, which considered risk of bias, inconsistency, indirectness, imprecision, and publication bias [33]. All analyses were conducted using RevMan (Version 5.3; Cochrane Collaboration, Oxford, UK) and Stata (Version 17.0; Stata Corporation, College Station, TX, USA).

## Results

### Study inclusion

The study selection process is illustrated in Figure 1. Initially, we identified 1129 records from three databases. After eliminating 311 duplicates, 818 articles were screened based on their titles and abstracts. Of these, 783 articles were excluded for failing to meet the criteria of the meta-analysis. The full texts of the remaining 35 articles were subsequently reviewed by two independent authors, resulting in the exclusion of 20 articles for various reasons (refer to Figure 1). Ultimately, 15 studies were included in the quantitative analysis [16–30].

### Summary of study characteristics

Table 1 presents the characteristics of the 15 cross-sectional studies included in this meta-analysis, published between 2007 and 2025, and conducted across various regions, including Spain, Japan, Canada, Taiwan (China), the United States, Korea, Poland, China, and multiple international centers. These studies collectively involved 569,856 children and adolescents, with participant ages ranging from 2 to 18 years and mean ages between 6.5 and 15.5 years. All studies employed a cross-sectional design, with obesity primarily defined using age- and sex-specific BMI cutoffs at the ≥ 95th percentile [19, 21, 24, 26, 27, 29, 30], international BMI standards [18, 20, 22, 23, 25], or country-specific thresholds (e.g., ≥25 kg/m^2^ in Korea, >23 kg/m^2^ in Taiwan) [16, 17, 28]. The number of obese children reported in each study varied widely, ranging from 130 to 14,452.

**Table 1:** Characteristics of the included studies

**Study**	**Country**	**Design**	**Participant characteristics**	**No. of participants**	**Age ranges (years)**	**Mean age (years)**	**Boy (%)**	**Definition of obesity**	**No. of children with obesity**	**Diagnosis of AR**	**No. of children with AR**	**Variables adjusted**
Garcia-Marcos, 2007	Spain	CS	School children	20,160	6∼7	6.5	NR	International BMI cut-offs	NR	ISAAC questionnaire (sneezing/runny nose + itchy/watery eyes)	1446	Age, sex, maternal smoking, siblings, exercise, Mediterranean diet score
Kusunoki, 2008	Japan	CS	School children	45,520	7∼15	10.9	50.3	BMI ≥ 95th percentile (age/sex-specific)	NR	ISAAC-based questionnaire (sneezing/runny nose + nasal obstruction/itching)	9888	Age, sex, birth order, other allergic diseases
Wang, 2010	Canada	CS	School children	8334	13∼14	13.4	46.7	International BMI cut-offs	330	ISAAC criteria (current rhinoconjunctivitis: sneezing/runny nose + itchy eyes)	1572	Age, sex, region, birthplace, ethnicity, maternal education, siblings, smoking, traffic exposure, pet ownership, acetaminophen use, physical activity, TV time
Tanaka, 2011	Japan	CS	School children	24,399	6∼15	10.0	49.2	BMI ≥ 95th percentile (age/sex-specific)	NR	ISAAC criteria (sneezing/nasal symptoms + itchy-watery eyes)	1854	Age, sex, region, siblings, household smoking, physical activity, parental history of allergies, parental education
Yao, 2011	Taiwan (China)	CS	School children	5351	4∼18	10.4	48.9	International BMI cut-offs	435	ISAAC criteria (current rhinitis = sneezing/runny/blocked nose in past 12 months)	2365	Age and sex
Mitchell, 2013	Multi-national (29 centers, 17 countries)	CS	School children	27,7534	6∼7 and 13∼14	6.5 and 13.5	NR	International BMI cut-offs	NR	ISAAC criteria (current rhinoconjunctivitis)	NR	Age, sex, region, language, income, physical activity, TV viewing, BMI measurement type
Sidell, 2013	USA	CS	School children	10,623	6∼17	12.2	51	BMI ≥ 95th percentile (age/sex-specific)	2776	ICD-9 code	NR	Age, sex, race/ethnicity, insurance status, geographic region
Weinmayr, 2014	Multi-country	CS	School children	10,652	8∼12	9.5	NR	International BMI cut-offs	1925	ISAAC questionnaire	NR	Age and sex
Sybilski, 2015	Poland	CS	School children	9231	6∼7 and 13∼14	6.5 and 13.5	51.3	BMI ≥ 95th percentile (age/sex-specific)	439	ISAAC questionnaire	2216	Age, sex, and urban/rural residence
Lin, 2015	Taiwan (China)	CS	High-school children	74,688	13∼15	14.0	50.7	BMI > 23 kg/m^2^	14,452	Physician-diagnosed + symptoms	16,720	Age, sex, parental education, environmental tobacco smoke
Lei, 2016	China	CS	Cluster-stratified random sampling from community	3327	2∼14	8.0	50	BMI ≥ 95th percentile (age/sex-specific)	417	Physician-diagnosed (ARIA criteria)	588	Age and sex
Lee, 2016	Korea	CS	Nationally representative survey (KYRBWS-VII)	75,643	13∼18	15.5	50	BMI ≥ 25 kg/m^2^ (Korea standard)	8926	Self-reported physician diagnosis	25,643	Age, sex, residence, family affluence scale, parental education, academic achievement, smoking, drinking
Han, 2016	USA	CS	Nationally representative survey	2358	6∼17	11.8	49.8	BMI ≥ 95th percentile (age/sex-specific)	825	Physician-diagnosed + symptoms + ≥1 positive IgE	295	Age, sex, race, income, cotinine, asthma, CRP
McArdle, 2024	USA	CS	Children attending otolaryngology clinic	406	2∼18	10.7	55.5	BMI ≥ 95th percentile (age/sex-specific)	130	Physician-diagnosed	NR	Age, sex, and comorbidities
Lee, 2025	Korea	CS	Nationally representative survey (KNHNES)	1630	13∼18	15.5	54.5	BMI ≥ 25 kg/m^2^ (Korea standard)	234	Self-reported physician diagnosis	374	Age and sex

Abbreviations: AR: Allergic rhinitis; ARIA: Allergic rhinitis and its impact on asthma; BMI: Body mass index; CRP: C-reactive protein; CS: Cross-sectional study; ICD-9: International Classification of Diseases, Ninth Revision; IgE: Immunoglobulin E; ISAAC: International Study of Asthma and Allergies in Childhood; KNHNES: Korea National Health and Nutrition Examination Survey; KYRBWS: Korea Youth Risk Behavior Web-Based Survey; NR: Not reported in the original publication: Values were not available and were not imputed by the authors.

AR was most frequently diagnosed using the ISAAC questionnaire in eight studies [18–23, 25, 26]. Additional diagnostic methods included physician diagnosis in six studies [16, 17, 27–30] and the use of International Classification of Disease codes in one study [24]. Some studies also integrated symptom criteria and/or objective measures, such as positive IgE tests [27, 30]. The number of AR cases reported across studies ranged from 295 to over 25,000. All studies adjusted for at least age and sex in their analyses, with many accounting for additional confounders, including household smoking, parental education, physical activity, environmental exposures (e.g., traffic or secondhand smoke), socioeconomic indicators, and other allergic conditions.

As illustrated in Table 2, the methodological quality of the included studies, assessed using the NOS, was high, with NOS scores ranging from seven to nine. Six studies achieved a full score of nine, indicating appropriate case and control selection, adequate adjustment for age, sex, and other confounders, as well as consistent exposure and outcome ascertainment methods [16, 18–21, 27]. The remaining nine studies scored seven or eight [17, 22–26, 28–30], primarily due to limitations in case definition, incomplete adjustment for additional confounders, or lack of representativeness. Despite variability in design details, all studies met the criteria for valid AR ascertainment and employed comparable assessment methods between groups, thereby supporting the reliability of the pooled estimates.

**Table 2:** Study quality evaluation via the modified Newcastle–Ottawa Scale

**Study**	**Adequate definition of cases**	**Representativeness of cases**	**Selection of controls**	**Definition of controls**	**Control for age and sex**	**Control for other confounders**	**Exposure ascertainment**	**Same methods for events ascertainment**	**Non-response rates**	**Total**
Garcia-Marcos, 2007	1	1	1	1	1	1	1	1	1	9
Kusunoki, 2008	1	1	1	1	1	1	1	1	1	9
Wang, 2010	1	1	1	1	1	1	1	1	1	9
Tanaka, 2011	1	1	1	1	1	1	1	1	1	9
Yao, 2011	1	1	1	1	1	0	1	1	1	8
Mitchell, 2013	1	1	1	1	1	1	1	1	0	8
Sidell, 2013	0	1	1	1	1	1	1	1	0	7
Weinmayr, 2014	1	1	1	1	1	0	1	1	0	7
Sybilski, 2015	1	1	1	1	1	0	1	1	0	7
Lin, 2015	1	1	1	1	1	1	1	1	1	9
Lei, 2016	1	1	1	1	1	0	1	1	1	8
Lee, 2016	0	1	1	1	1	1	1	1	1	8
Han, 2016	1	1	1	1	1	1	1	1	1	9
McArdle, 2024	1	0	1	1	1	1	1	1	0	7
Lee, 2025	0	1	1	1	1	0	1	1	1	7

### Association between obesity and AR in children

Five studies reported the association between obesity and AR in boys and girls separately [18, 19, 23, 27, 28], while two studies examined this association in two independent age groups [23, 26]. Consequently, these stratified datasets were independently included in the meta-analysis, resulting in a total of 23 datasets. This methodology permitted more nuanced subgroup analyses.

**Figure 2.**
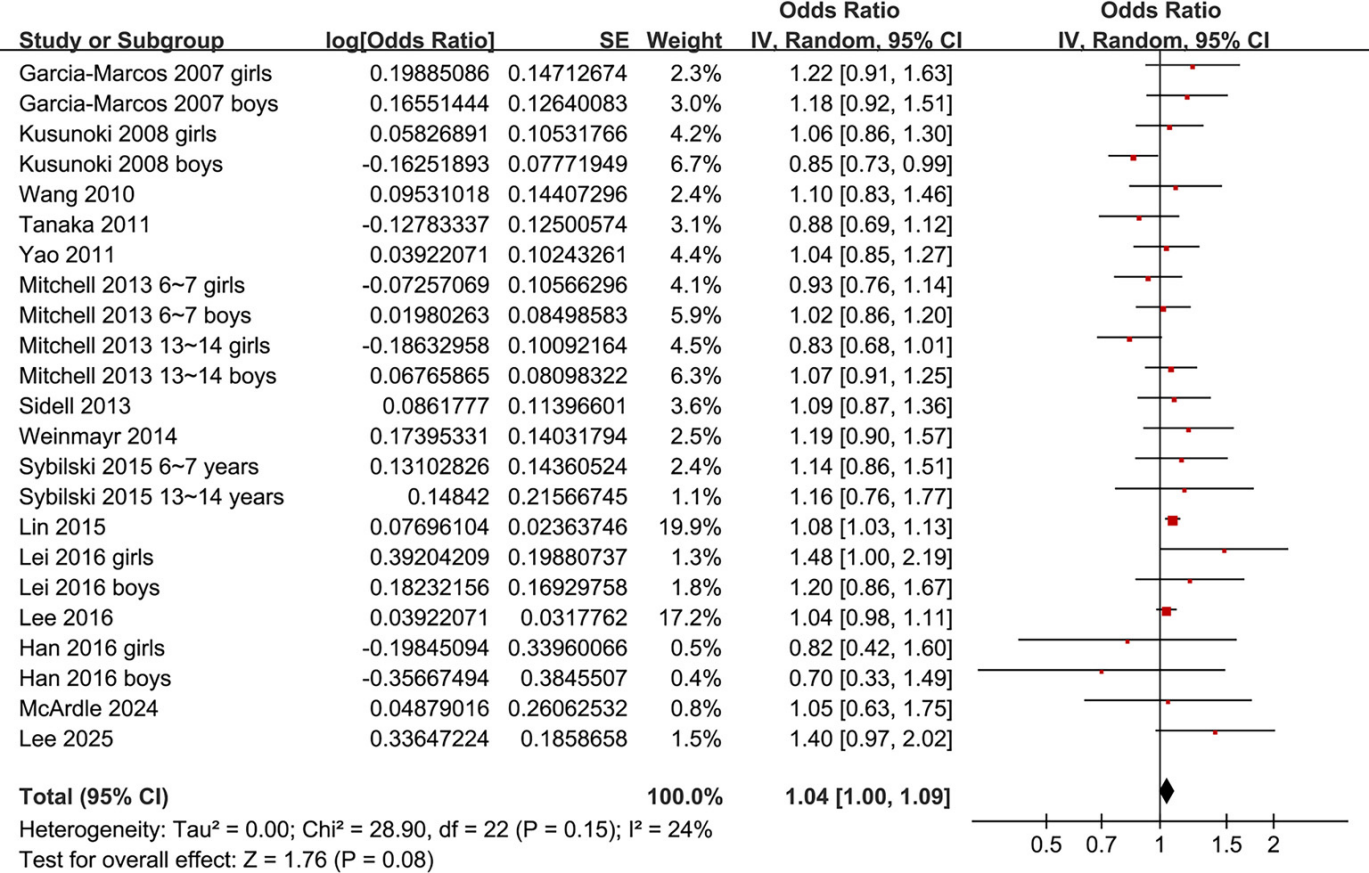
**Association between obesity and allergic rhinitis (AR) in children.** Forest plot of 23 datasets assessing the association between obesity and AR in children. Pooled analysis showed no significant association (OR: 1.04, 95% CI: 1.00–1.09, *P* ═ 0.08), with low heterogeneity (*I*^2^ ═ 24%). Abbreviations: OR: Odds ratio; CI: Confidence interval; SE: Standard error; IV: Inverse variance; df: Degrees of freedom.

Pooled results indicated that obesity was not significantly associated with AR in children (adjusted OR: 1.04, 95% CI: 1.00–1.09, *P* ═ 0.08; Figure 2), with mild heterogeneity observed (*P* for Cochrane *Q* test = 0.15; *I*^2^ ═ 24%). A sensitivity analysis employing the REML model yielded similar findings (adjusted OR: 1.04, 95% CI: 1.00–1.09, *P* ═ 0.07; *I*^2^ ═ 28%; Figure S1). Furthermore, when we combined sex-stratified datasets into a single effect size per study, the results mirrored those of the main analysis (adjusted OR: 1.05, 95% CI: 1.00–1.09, *P* ═ 0.05; *I*^2^ ═ 14%; Figure S2), suggesting that potential clustering within studies did not significantly impact our conclusions. Sensitivity analysis, omitting one dataset at a time, demonstrated consistent results (OR: 1.03–1.06, *P* all > 0.05). Additionally, limiting sensitivity analysis to studies with an NOS score of ≥ 8 yielded similar outcomes (adjusted OR: 1.03, 95% CI: 0.97–1.08, *P* ═ 0.36; *I*^2^ ═ 36%).

**Figure 3.**
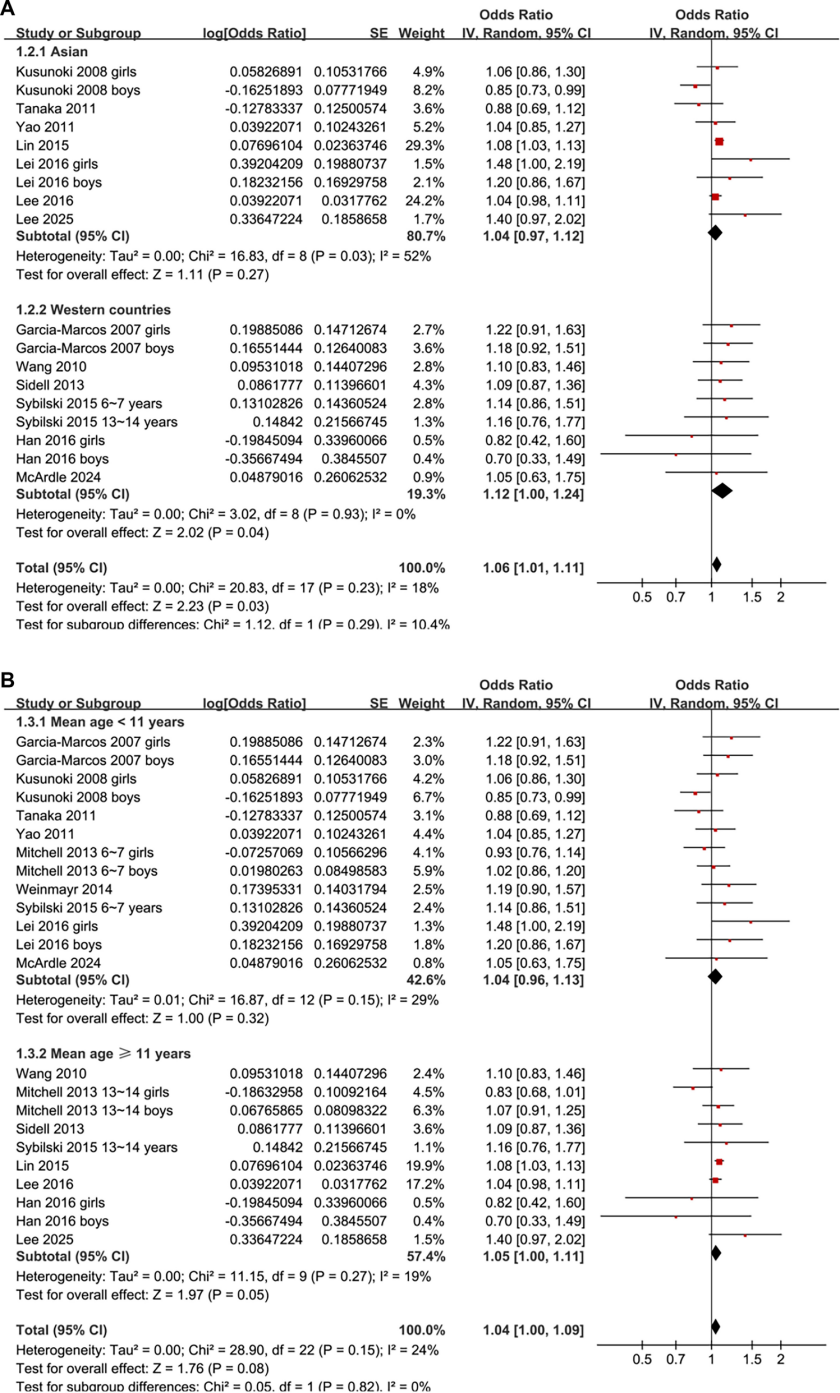
**Subgroup analyses of the association between obesity and allergic rhinitis (AR) in children by region and age.** (A) Forest plot of studies stratified by geographic region (Asian vs Western countries). Obesity was associated with AR only in children from Western countries (OR: 1.12, 95% CI: 1.00–1.24, *P* ═ 0.04; *I*^2^ ═ 0%), but not in Asian children (OR: 1.04, 95% CI: 0.97–1.12, *P* ═ 0.27; *I*^2^ ═ 52%). (B) Forest plot of studies stratified by mean age (< 11 vs ≥ 11 years), showing no significant subgroup differences (*P* for subgroup difference = 0.82). Note: The regional subtotal in Figure 3A excludes multinational datasets that could not be uniquely assigned to either Asian or Western categories, and therefore does not equal the overall total reported in Figure 2. Abbreviations: OR: Odds ratio; CI: Confidence interval; SE: Standard error; IV: Inverse variance; df: Degrees of freedom.

**Figure 4.**
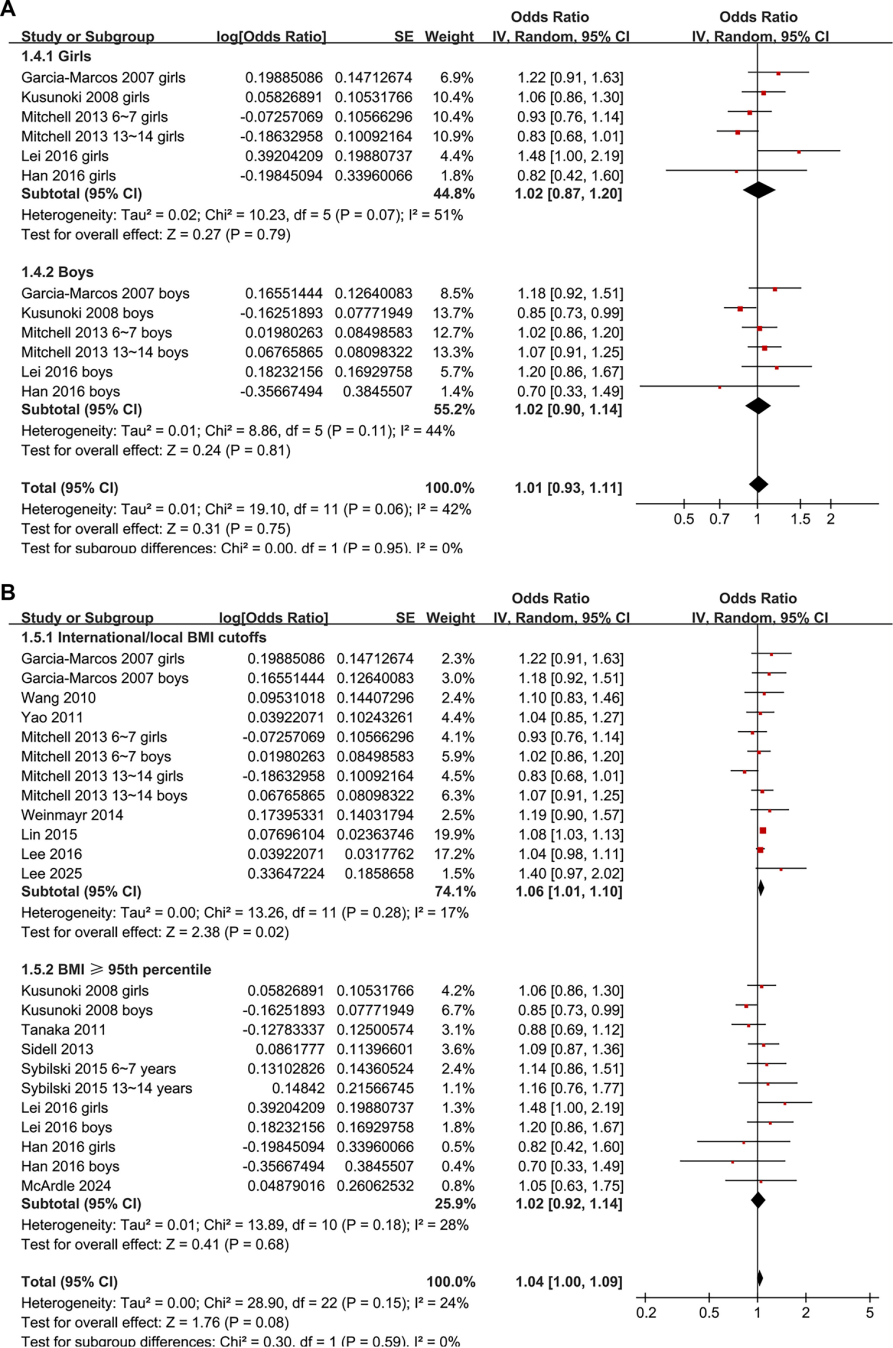
**Subgroup analyses of the association between obesity and allergic rhinitis (AR) in children by sex and obesity definition.** (A) Forest plot of studies stratified by sex, showing no significant subgroup differences between girls and boys (*P* ═ 0.95); (B) Forest plot of studies stratified by obesity definition. A significant association was observed in studies using national/international BMI cutoffs (OR: 1.06, 95% CI: 1.01–1.10, *P* ═ 0.02; *I*^2^ ═ 17%), but not in those using BMI ≥ 95th percentile (OR: 1.02, 95% CI: 0.92–1.14, *P* ═ 0.68; *I*^2^ ═ 28%). Abbreviations: OR: Odds ratio; CI: Confidence interval; SE: Standard error; IV: Inverse variance; df: Degrees of freedom; BMI: Body mass index.

Subgroup analyses revealed a significant association between obesity and AR in children from Western countries (OR: 1.12, 95% CI: 1.00–1.24, *P* ═ 0.04; *I*^2^ ═ 0%), but not in children from Asian countries (OR: 1.04, 95% CI: 0.97–1.12, *P* ═ 0.27; *I*^2^ ═ 52%; Figure 3A). Similar results were observed across studies of children with mean ages < or ≥ 11 years (*P* for subgroup difference = 0.82; Figure 3B) and between girls and boys (*P* for subgroup difference = 0.95; Figure 4A). A significant association between obesity and AR was noted in studies defining obesity based on national or international BMI cutoffs (OR: 1.06, 95% CI: 1.01–1.10, *P* ═ 0.02; *I*^2^ ═ 17%), but not in studies using age- and sex-specific 95th percentile definitions (OR: 1.02, 95% CI: 0.92–1.14, *P* ═ 0.68; *I*^2^ ═ 28%; Figure 4B).

**Figure 5.**
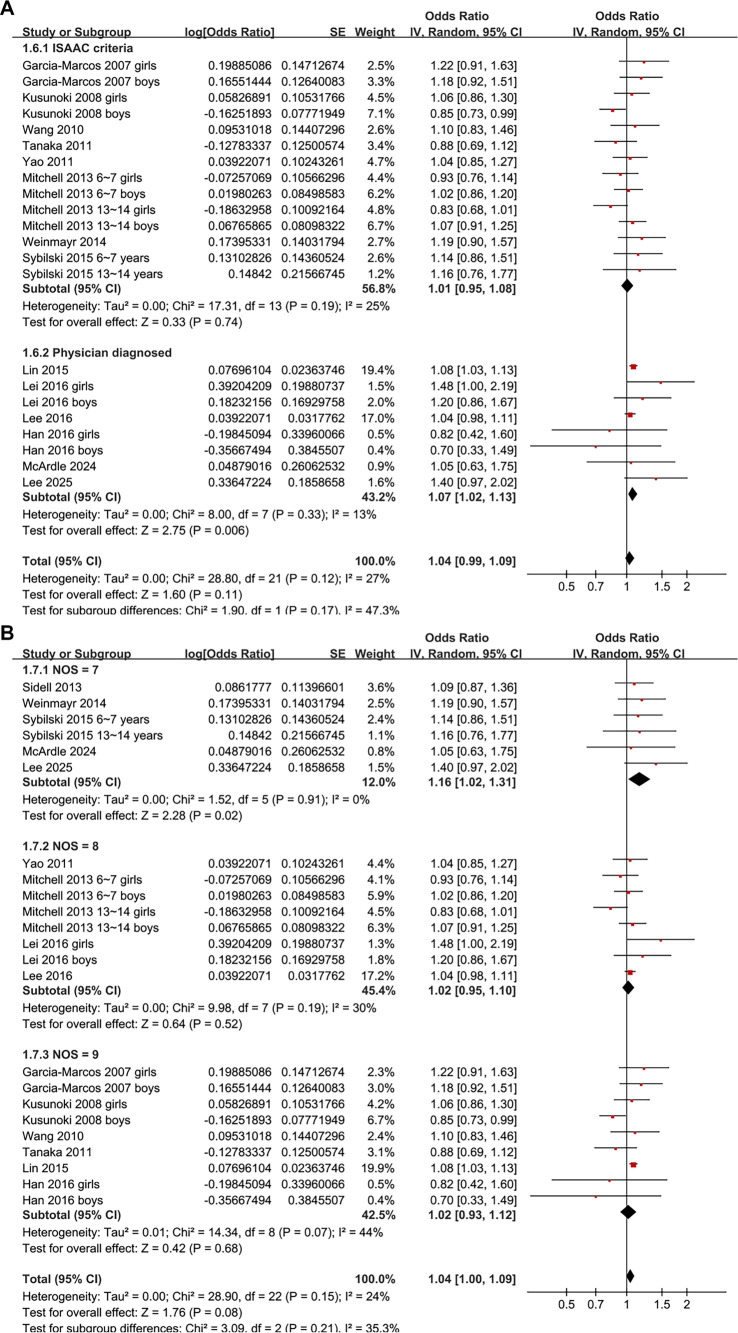
**Subgroup analyses of the association between obesity and allergic rhinitis (AR) in children by diagnostic method and study quality.** (A) Obesity was significantly associated with physician-diagnosed AR (OR: 1.07, 95% CI: 1.02–1.13), but not with AR defined by the ISAAC questionnaire; (B) A significant association was observed in studies with NOS = 7, but not in those with NOS = 8 or 9. Abbreviations: OR: Odds ratio; CI: Confidence interval; SE: Standard error; IV: Inverse variance; df: Degrees of freedom; BMI: Body mass index; ISAAC: International Study of Asthma and Allergies in Childhood; NOS: Newcastle–Ottawa Scale.

Notably, a significant association between obesity and AR was found in studies where AR was physician-diagnosed (OR: 1.07, 95% CI: 1.02–1.13, *P* ═ 0.006; *I*^2^ ═ 13%), but not in studies using the ISAAC questionnaire for diagnosis (OR: 1.01, 95% CI: 0.95–1.08, *P* ═ 0.74; *I*^2^ ═ 25%; Figure 5A). Furthermore, a significant association was observed in studies with NOS = 7 (OR: 1.16, 95% CI: 1.02–1.31, *P* ═ 0.02; *I*^2^ ═ 0%), but not in studies with NOS = 8 (OR: 1.02, 95% CI: 0.95–1.10, *P* ═ 0.52; *I*^2^ ═ 30%) or NOS = 9 (OR: 1.02, 95% CI: 0.93–1.12, *P* ═ 0.68; *I*^2^ ═ 44%; Figure 5B).

Finally, univariate meta-regression analysis indicated that mean age, proportion of males, and NOS did not significantly influence the association between obesity and AR (*P* all > 0.05; Table 3). According to the GRADE framework, the certainty of the evidence was rated as low, reflecting the cross-sectional nature of the studies and the imprecision of effect estimates, although concerns regarding heterogeneity, indirectness, and publication bias were not deemed serious (Table S1).

**Table 3:** Results of univariate meta-regression analysis

**Variables**	**OR for the association between obesity and AR**			
	**Coefficient**	**95% CI**	***P* values**	**Adjusted R^2^**
Mean age (years)	−0.0022	−0.0195 to 0.0152	0.80	0%
Male (%)	−0.0015	−0.1665 to 0.1636	0.99	0%
NOS	−0.047	−0.126 to 0.033	0.24	0%

Abbreviations: OR: Odds ratio; CI: Confidence interval; AR: Allergic rhinitis; NOS: Newcastle-Ottawa Scale.

### Publication bias

**Figure 6.**
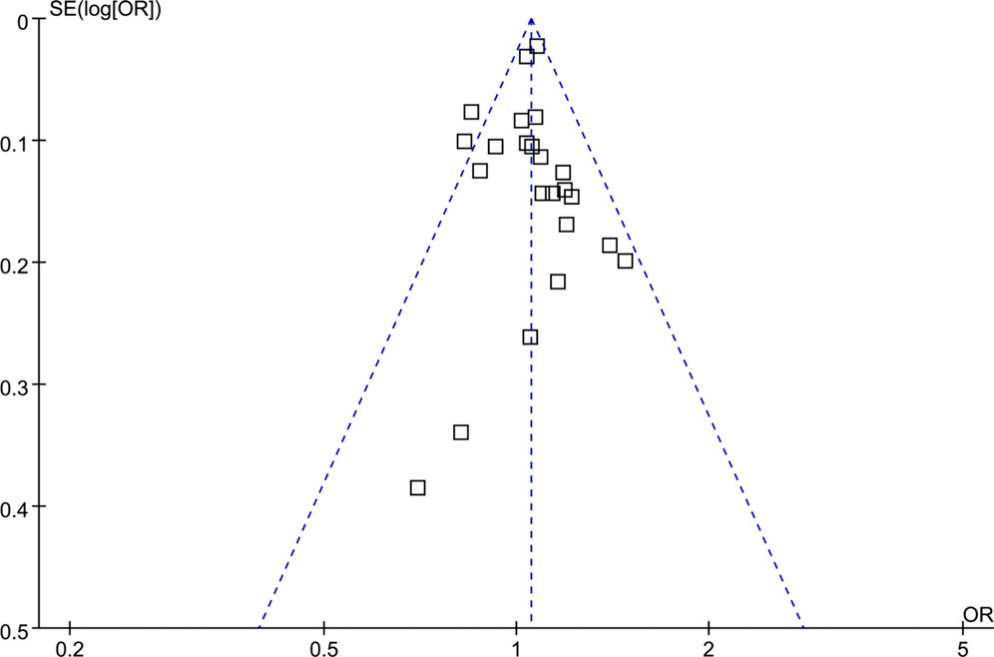
**Funnel plot of the association between obesity and allergic rhinitis (AR) in children.** The plot appeared symmetrical, and Egger’s test showed no evidence of publication bias (*P* ═ 0.43).

Funnel plots illustrating the meta-analyses of the association between obesity and AR in children are presented in Figure 6. The symmetry of the plots indicates a low risk of publication bias. Additionally, Egger’s test revealed no significant evidence of publication bias (*P* ═ 0.43).

## Discussion

This meta-analysis, encompassing over half a million children across 15 studies, found no significant overall association between obesity and AR after adjusting for age, sex, and other relevant covariates. However, subgroup analyses revealed a stronger association in children from Western countries, in studies utilizing national or international BMI cutoffs, in studies with physician-diagnosed AR, and in those with an NOS score of 7. These findings suggest that the relationship between obesity and AR in children may vary according to geographic region, definitions of obesity and AR, and the quality of the study design.

Our results refine and contextualize previous syntheses. An early meta-analysis published in 2020 reported a modest but significant association between obesity or overweight and AR in children (OR: 1.09; 95% CI: 1.04–1.14). However, that analysis combined children and adults and included both overweight and obese categories, potentially inflating the pooled estimates [39]. More recently, Yeo et al. [40] conducted a large-scale meta-analysis that included both children and adults with abnormal BMI (overweight and obesity) and found no significant association with AR in any subgroup. Our meta-analysis diverges from these prior studies by exclusively including children and adolescents, focusing solely on obesity (not overweight), requiring multivariate adjustments (at least for age and sex), and performing detailed subgroup analyses to explore sources of heterogeneity. Compared to earlier work, this study provides more nuanced insights into specific subgroups where the association may be more pronounced or absent.

The stronger association observed in Western countries may be attributed to several factors. Lifestyle and dietary patterns in Western populations, including higher consumption of processed foods, sedentary behaviors, and increased prevalence of obesity-related metabolic inflammation, could amplify immune dysregulation and heighten susceptibility to AR [41]. Environmental exposures also differ; Western children may experience greater exposure to indoor allergens, pollutants, or urban environments that interact with obesity-related inflammation to exacerbate airway responses. In contrast, Asian countries may report weaker associations partly due to stricter diagnostic thresholds for AR, disparities in healthcare access, and cultural variations in healthcare-seeking behavior, leading to underdiagnosis [42]. Genetic differences, distinct microbiome profiles, and protective early-life exposures (e.g., traditional diets and lower prevalence of early-life obesity) may also attenuate the observed association in Eastern populations. Collectively, these factors may explain the regional heterogeneity observed in our subgroup analysis. The definition of obesity appeared to influence the observed relationship with AR. Studies utilizing fixed national or international BMI cutoffs (e.g., ≥ 25 kg/m^2^) demonstrated a significant association, while those employing age- and sex-specific ≥ 95th percentile cutoffs did not. This discrepancy may reflect measurement inconsistencies or differences in the populations captured by each approach. Fixed cutoffs may classify more children as obese in certain populations, particularly adolescents where BMI can plateau, thereby increasing statistical power to detect associations [43]. Conversely, the percentile-based method may misclassify children near the threshold and dilute the effect [43]. Our findings also indicate that AR diagnostic methods influence observed associations. Studies using physician-diagnosed AR reported a significant association with obesity, while those using the ISAAC questionnaire did not. Physician diagnosis may incorporate clinical judgment, symptom severity, and objective evidence (e.g., IgE testing), providing a more accurate definition of AR [44]. The ISAAC questionnaire, while validated, may be more susceptible to recall bias and variability in parental reporting [44]. This discrepancy highlights the need for standardized and objective AR diagnosis in future epidemiologic studies. Notably, the association was strongest in studies with an NOS score of 7 but was not significant in studies with scores of 8 or 9. However, the between-subgroup *P* value was not significant, and meta-regression did not support NOS score as a moderator. Given that all included studies were rated as high quality (NOS ≥ 7), this apparent paradox is unlikely to reflect a genuine quality gradient and may instead arise from chance variation or sample characteristics. Thus, while subgroup patterns are identified, our overall conclusion is grounded in consistently high-quality evidence across all studies.

Several biological mechanisms may underlie the potential link between obesity and AR in children. Adipose tissue functions as an endocrine organ that secretes pro-inflammatory cytokines such as tumor necrosis factor-alpha, interleukin-6, and leptin, which can amplify systemic inflammation and immune dysregulation [45]. These mediators may alter the balance between T-helper 1 and T-helper 2 immune responses, promote eosinophilic inflammation, and compromise epithelial barrier integrity in the airway mucosa [46]. Obesity-related changes in lung mechanics, such as reduced functional residual capacity and altered nasal airflow, may further predispose obese children to airway inflammation and allergen sensitization [47]. Recent experimental work has demonstrated that modulation of the toll-like receptor 4/mitogen-activated protein kinase/nuclear factor kappa B signaling pathway plays a key role in AR pathogenesis, with chlorogenic acid shown to exert therapeutic effects through this mechanism [48]. These immunologic and physiologic pathways provide a plausible foundation for the observed association between obesity and AR, particularly in children with coexisting atopic conditions.

This study has several strengths. It represents a comprehensive and up-to-date synthesis focusing exclusively on childhood obesity and AR in children. By requiring multivariable adjustment (at least for age and sex), our analysis minimizes confounding and complements recent broader meta-analyses that combined children and adults. We applied stringent inclusion criteria, conducted extensive subgroup analyses, and assessed heterogeneity and publication bias using established methods. Nonetheless, several limitations should be acknowledged. First, although our protocol was registered in PROSPERO, this occurred after the completion of the database search, meaning the registration was not strictly prospective. While the review protocol was defined in advance and adhered to, post-hoc registration may raise concerns about selective reporting and should be considered when interpreting the subgroup findings. Second, the cross-sectional design of all included studies precludes causal inference; reverse causation remains possible, where AR or its treatment influences physical activity and weight [49]. Although our inclusion criteria permitted longitudinal designs, no eligible cohort or case-control studies were identified, limiting the evidence base to cross-sectional studies. Third, the lack of individual participant data restricted our ability to explore dose-response relationships, detailed age stratification (e.g., preschoolers vs adolescents), or coexisting conditions such as asthma. Additionally, heterogeneity in definitions of obesity and AR may have influenced the findings, and residual confounding from unmeasured factors—such as diet, vitamin D status, genetic susceptibility, or allergen exposure—cannot be excluded. Furthermore, the majority of studies relied on self-reported or questionnaire-based data, which may be subject to recall and misclassification biases. Another limitation is that some included studies reported stratified results by age or sex, which we treated as independent datasets to facilitate more detailed subgroup analyses. While this is a common approach in meta-analyses, it may not fully account for within-study clustering. Nevertheless, as only a few studies contributed stratified data and leave-one-out sensitivity analyses showed stable findings, the potential influence on variance estimates is likely minimal. Moreover, the subgroup and meta-regression analyses were based on study-level covariates rather than individual participant data, which may introduce ecological bias. Therefore, the absence of significant moderator effects should be interpreted with caution. Furthermore, we could not evaluate adjustment depth (e.g., number or type of covariates included) through meta-regression due to substantial variability across studies. This variability limits our ability to formally assess whether differences in confounder adjustment contributed to heterogeneity. Excluding overweight individuals may also limit the generalizability of findings, though it strengthens the specificity of our exposure definition. Finally, our restriction to English-language, peer-reviewed publications and the exclusion of grey literature may have introduced language or publication bias. Although Egger’s test did not indicate significant publication bias (*P* ═ 0.43), this possibility cannot be entirely ruled out.

Clinically, these findings suggest that obesity may contribute to AR risk in specific pediatric populations, particularly in Western countries or when using standardized diagnostic criteria. However, given the non-significant overall association and the cross-sectional design of all included studies, causal inferences cannot be made. Therefore, while preventive strategies against childhood obesity remain vital for various health outcomes, their potential to reduce allergic burden requires confirmation in longitudinal and interventional studies. Additionally, the modest effect sizes observed may underscore the need for multifactorial approaches in AR prevention and management. Future research should prioritize large-scale, prospective cohort studies with standardized definitions of both obesity and AR, detailed phenotyping of allergic disease, and adjustment for a broader range of confounders. Investigating the role of obesity interventions in AR prevention or symptom control, as well as exploring underlying molecular mechanisms through systems biology or biomarker studies, could offer novel insights.

## Conclusion

In conclusion, childhood obesity was not significantly associated with the overall risk of AR. However, subgroup analyses revealed significant associations in studies utilizing national and international BMI cut-offs and those employing physician-diagnosed AR criteria. These findings suggest that methodological differences and population context may impact observed associations, highlighting the necessity for longitudinal studies to confirm these results.

### GAMER statement

The authors declare that no generative AI tools were utilized in the writing, editing, preparation of figures and tables, or management of references for this manuscript.

## Supplemental data

Supplemental data are available at the following link: https://www.bjbms.org/ojs/index.php/bjbms/article/view/12982/4010.

## Data Availability

All data generated or analyzed during this study are included in this published article.

## References

[1] Licari A, Magri P, De Silvestri A, Giannetti A, Indolfi C, Mori F (2023). Epidemiology of allergic rhinitis in children: a systematic review and meta-analysis. J Allergy Clin Immunol Pract.

[2] Okano M, Fujieda S, Gotoh M, Kurono Y, Matsubara A, Ohta N (2023). Executive summary: Japanese guidelines for allergic rhinitis 2020. Allergol Int.

[3] Gutowska-Ślesik J, Samoliński B, Krzych-Fałta E (2023). The increase in allergic conditions based on a review of literature. Postepy Dermatol Alergol.

[4] Zhang X, Zhang M, Sui H, Li C, Huang Z, Liu B (2023). Prevalence and risk factors of allergic rhinitis among Chinese adults: a nationwide representative cross-sectional study. World Allergy Organ J.

[5] Alblewi SMS, Alenazi LM, Alshahrani RS, Alharbi RT, Alotaibi NA, Albalawi HMD (2024). Prevalence of allergic rhinitis and its impact on quality of life among pediatric patients in Tabuk, Saudi Arabia. Oman Med J.

[6] Blaiss MS, Hammerby E, Robinson S, Kennedy-Martin T, Buchs S (2018). The burden of allergic rhinitis and allergic rhinoconjunctivitis on adolescents: a literature review. Ann Allergy Asthma Immunol.

[7] Steiner UC, Bachmann LM, Soyka MB, Regenass S, Steinegger L, Probst E (2018). Relationship between rhinitis, asthma, and eczema and the presence of sensitization in young swiss adults. Allergy Rhinol (Providence).

[8] Wang DY (2005). Risk factors of allergic rhinitis: genetic or environmental?. Ther Clin Risk Manag.

[9] Murrison LB, Brandt EB, Myers JB, Hershey GKK (2019). Environmental exposures and mechanisms in allergy and asthma development. J Clin Invest.

[10] Hampl SE, Hassink SG, Skinner AC, Armstrong SC, Barlow SE, Bolling CF (2023). Clinical practice guideline for the evaluation and treatment of children and adolescents with obesity. Pediatrics.

[11] Jebeile H, Kelly AS, O’Malley G, Baur LA (2022). Obesity in children and adolescents: epidemiology, causes, assessment, and management. Lancet Diabetes Endocrinol.

[12] Marcus C, Danielsson P, Hagman E (2022). Pediatric obesity-long-term consequences and effect of weight loss. J Intern Med.

[13] Chung ST, Krenek A, Magge SN (2023). Childhood obesity and cardiovascular disease risk. Curr Atheroscler Rep.

[14] Raj D, Kabra SK, Lodha R (2014). Childhood obesity and risk of allergy or asthma. Immunol Allergy Clin North Am.

[15] Voltan C, Concer F, Pecoraro L, Pietrobelli A, Piacentini G, Zaffanello M (2024). Exploring the complex interplay of obesity, allergic diseases, and sleep-disordered breathing in children. Children (Basel).

[16] Lin MH, Hsieh CJ, Caffrey JL, Lin YS, Wang IJ, Ho WC (2015). Fetal growth, obesity, and atopic disorders in adolescence: a retrospective birth cohort study. Paediatr Perinat Epidemiol.

[17] Lee HJ, Jeon YH (2025). The effect of environmental factors, health behaviors, and psychosocial aspects on allergic diseases in Korean adolescents. Medicina (Kaunas).

[18] Garcia-Marcos L, Canflanca IM, Garrido JB, Varela AL, Garcia-Hernandez G, Guillen Grima F (2007). Relationship of asthma and rhinoconjunctivitis with obesity, exercise and Mediterranean diet in Spanish schoolchildren. Thorax.

[19] Kusunoki T, Morimoto T, Nishikomori R, Heike T, Ito M, Hosoi S (2008). Obesity and the prevalence of allergic diseases in schoolchildren. Pediatr Allergy Immunol.

[20] Wang HY, Pizzichini MM, Becker AB, Duncan JM, Ferguson AC, Greene JM (2010). Disparate geographic prevalences of asthma, allergic rhinoconjunctivitis and atopic eczema among adolescents in five Canadian cities. Pediatr Allergy Immunol.

[21] Tanaka K, Miyake Y, Arakawa M, Sasaki S, Ohya Y (2011). U-shaped association between body mass index and the prevalence of wheeze and asthma, but not eczema or rhinoconjunctivitis: the ryukyus child health study. J Asthma.

[22] Yao TC, Ou LS, Yeh KW, Lee WI, Chen LC, Huang JL (2011). Associations of age, gender, and BMI with prevalence of allergic diseases in children: PATCH study. J Asthma.

[23] Mitchell EA, Beasley R, Björkstén B, Crane J, García-Marcos L, Keil U (2013). The association between BMI, vigorous physical activity and television viewing and the risk of symptoms of asthma, rhinoconjunctivitis and eczema in children and adolescents: ISAAC phase three. Clin Exp Allergy.

[24] Sidell D, Shapiro NL, Bhattacharyya N (2013). Obesity and the risk of chronic rhinosinusitis, allergic rhinitis, and acute otitis media in school-age children. Laryngoscope.

[25] Weinmayr G, Forastiere F, Büchele G, Jaensch A, Strachan DP, Nagel G (2014). Overweight/obesity and respiratory and allergic disease in children: international study of asthma and allergies in childhood (ISAAC) phase two. PLoS One.

[26] Sybilski AJ, Raciborski F, Lipiec A, Tomaszewska A, Lusawa A, Furmanczyk K (2015). Obesity—a risk factor for asthma, but not for atopic dermatitis, allergic rhinitis and sensitization. Public Health Nutr.

[27] Han YY, Forno E, Gogna M, Celedon JC (2016). Obesity and rhinitis in a nationwide study of children and adults in the United States. J Allergy Clin Immunol.

[28] Lee KS, Rha YH, Oh IH, Choi YS, Choi SH (2016). Socioeconomic and sociodemographic factors related to allergic diseases in Korean adolescents based on the seventh Korea youth risk behavior web-based survey: a cross-sectional study. BMC Pediatr.

[29] Lei Y, Yang H, Zhen L (2016). Obesity is a risk factor for allergic rhinitis in children of Wuhan (China). Asia Pac Allergy.

[30] McArdle E, Cummins M, Shetty S, Chaiban R, Ramadan HH, Makary CA (2024). The association between obesity and the unified airway in children. Ear Nose Throat J.

[31] Page MJ, McKenzie JE, Bossuyt PM, Boutron I, Hoffmann TC, Mulrow CD (2021). The PRISMA 2020 statement: an updated guideline for reporting systematic reviews. BMJ.

[32] Page MJ, Moher D, Bossuyt PM, Boutron I, Hoffmann TC, Mulrow CD (2021). PRISMA 2020 explanation and elaboration: updated guidance and exemplars for reporting systematic reviews. BMJ.

[33] Higgins J, Thomas J, Chandler J, Cumpston M, Li T, Page M, et al.

[34] Wells GA, Shea B, O’Connell D, Peterson J, Welch V, Losos M, et al. http://www.ohri.ca/programs/clinical/_epidemiology/oxford.asp.

[35] Herzog R, Álvarez-Pasquin MJ, Díaz C, Del Barrio JL, Estrada JM, Gil Á (2013). Are healthcare workers’ intentions to vaccinate related to their knowledge, beliefs and attitudes? A systematic review. BMC Public Health.

[36] Kim K, Shin S, Kim S, Lee E (2023). The relation between ehealth literacy and health-related behaviors: systematic review and meta-analysis. J Med Internet Res.

[37] Higgins JP, Thompson SG (2002). Quantifying heterogeneity in a meta-analysis. Stat Med.

[38] Egger M, Davey Smith G, Schneider M, Minder C (1997). Bias in meta-analysis detected by a simple, graphical test. BMJ.

[39] Zhou J, Luo F, Han Y, Lou H, Tang X, Zhang L (2020). Obesity/overweight and risk of allergic rhinitis: a meta-analysis of observational studies. Allergy.

[40] Yeo BSY, Guan EJ, Ng K, Lim YS, Goh RTH, Liu X (2025). Association of abnormal body weight and allergic rhinitis—a systematic review and meta-analysis. Clin Exp Allergy.

[41] Stefani C, Pecoraro L, Flodmark CE, Zaffanello M, Piacentini G, Pietrobelli A (2023). Allergic diseases and childhood obesity: a detrimental link?. Biomedicines.

[42] Chong SN, Chew FT (2018). Epidemiology of allergic rhinitis and associated risk factors in Asia. World Allergy Organ J.

[43] Zhang X, Liu J, Ni Y, Yi C, Fang Y, Ning Q (2024). Global prevalence of overweight and obesity in children and adolescents: a systematic review and meta-analysis. JAMA Pediatr.

[44] Wise SK, Damask C, Roland LT, Ebert C, Levy JM, Lin S (2023). International consensus statement on allergy and rhinology: allergic rhinitis—2023. Int Forum Allergy Rhinol.

[45] Clemente-Suárez VJ, Redondo-Flórez L, Beltrán-Velasco AI, Martín-Rodríguez A, Martínez-Guardado I, Navarro-Jiménez E (2023). The role of adipokines in health and disease. Biomedicines.

[46] Gauthier M, Kale SL, Ray A (2025). T1-T2 interplay in the complex immune landscape of severe asthma. Immunol Rev.

[47] Reyes-Angel J, Kaviany P, Rastogi D, Forno E (2022). Obesity-related asthma in children and adolescents. Lancet Child Adolesc Health.

[48] Xu X, Wang L, Wu G, Li X (2025). Therapeutic effects of chlorogenic acid on allergic rhinitis through TLR4/MAPK/NF-κB pathway modulation. Biomol Biomed.

[49] Park J, Park JH, Choi J, Kim TH (2020). Association between allergic rhinitis and regular physical activity in adults: a nationwide cross-sectional study. Int J Environ Res Public Health.

